# Evaluation of corneal dendritic cell density and subbasal nerve density in contact lens wearers using IVCM: A systematic review and meta-analysis

**DOI:** 10.3389/fmed.2023.1149803

**Published:** 2023-03-13

**Authors:** Rongjie Guo, Jiaxuan Jiang, Yanan Zhang, Qi Liang, Taige Chen, Kai Hu

**Affiliations:** Department of Ophthalmology, The Affiliated Drum Tower Hospital, Medical School of Nanjing University, Nanjing, China

**Keywords:** contact lens (CL), IVCM, dendritic cells, nerve, corneal

## Abstract

**Purpose:**

To evaluate the subclinical changes in corneal dendritic cell density (CDCD) and corneal subbasal nerve density (CSND) in asymptomatic contact lens (CL) wearers.

**Methods:**

Databases including PubMed, Scopus, Web of Science, and Cochrane Central Register of Controlled Trials were searched for trials and studies reporting the changes of corneal CDCD and CSND in contact lens wearers published until 25 June 2022. PRISMA guidelines as well as recommended meta-analysis practices were followed. Meta-analysis was conducted using RevMan V.5.3 software.

**Results:**

After the screening, 10 studies with 587 eyes of 459 participants were included. Seven studies reported the data of CDCD. Compared with the control group, CDCD in the CL wearers was higher (18.19, 95% CI 18.8–27.57, *p* = 0.0001). Type of *in vivo* confocal microscopy (IVCM), wear duration, and frequency of lens change were sources of heterogeneity. The difference in CSND between CL wearers and the control group was insignificant, and subgroup analysis did not reveal a source of heterogeneity.

**Conclusion:**

Overall, CDCD increased in CL wears, while CSND did not show significant differences. IVCM is a feasible tool to assess subclinical changes in CL wearers.

## Introduction

Contact lenses (CL) are an essential part of vision care worldwide. It is commonly used to correct refraction and slow the progression of myopia. However, up to 75% of CL wearers reported eye discomfort, which can lead to CL intolerance (1, 2). A significant number of CL wearers are clinically diagnosed with dry eye disease (DED) (2). Asymptomatic contact lens wearers refer to CL wearers who have not been clinically diagnosed with CL-related diseases such as DED and who have no subjective discomfort. Subclinical changes in asymptomatic contact lens wearers still require attention. Previous studies have demonstrated that wearing contact lenses leads to changes in the corneal nerves and increase in infiltrating immune cells (3, 4). The alteration in the corneal nerves is responsible for the symptom of eye discomfort (5), and the increased immune cells reflect rising levels of inflammation on the ocular surface (6). Changes in the corneal nerves and inflammation levels are core factors in the pathogenesis of DED and mediate the development of CL-related DED (7). Thus, the subclinical corneal changes of CL wearers and their relation to CL discomfort requires in-depth investigation.

As a non-invasive method, in vivo confocal microscopy (IVCM) has become increasingly important in diagnosing ocular and systemic diseases. Due to its ease of operation and 800× magnification of living cell structures, IVCM allows clinicians to observe the eye at the cellular level under *in vivo* conditions (8). In recent years, studies using IVCM have focused on assessing the cornea’s neuronal changes and inflammatory states. Studies in multiple diseases have demonstrated the alteration in the corneal nerves and increased immune cells, such as keratitis (9) and corneal dystrophies (10).

Although several studies have investigated the effects of CL on the ocular surface using IVCM, divergent results were yielded. Multiple clinical trials have examined the effects of CL wearing on the corneal nerve density; however, the field has yet to reach a consensus. Therefore, we conducted a meta-analysis of clinical trials to evaluate the effects of CL on the cornea, including corneal subbasal nerve density (CSND) and corneal dendritic cell density (CDCD).

## Methods

### Search methods

The following databases were searched for studies published up to 25 June 2022: Pubmed, Scopus, Web of Science, and Cochrane Central Register of Controlled Trials, using these search strategies (Contact Lens OR Lens, Contact OR Lenses, Contact) and (in vivo confocal microscopy OR confocal microscopy OR IVCM).

### Eligibility criteria for considering studies

Articles were included if they reported corneal dendritic cell density (CDCD) and corneal sub-basal nerve density (CSND) detected by in vivo confocal microscopy (IVCM). Exclusion Criteria were as follows: (1) subjects included minors; (2) inappropriate article type: such as reviews, case reports, conference papers, editorials, short surveys, or letters; (3) published not in English; (4) studies only contained cells and animal experiments; (5) subjects with systemic or ocular diseases or surgery history.

### Data collection

Search results from all electronic databases were exported to Endnote X9 reference management software for screening. The titles and abstracts of the articles were independently evaluated by two reviewers (RG and JJ). In cases of disagreements, full texts of articles were screened, and ambiguity was solved by discussion or consulting a third reviewer (KH). Data from included studies were extracted by a single reviewer (RG) in Microsoft Excel and checked by a second reviewer (JJ). Extracted information includes: the first author of the article, year of publication, country of the first author, journal of publication, number of patients, sample size (eyes), age of patients, gender of patients, type of IVCM systems, CL type, CL wearing duration, the mean and standard deviation of CDCD, and CSND.

### Risk of bias assessment

The studies included in this research consisted of case-control studies and cross-sectional studies. The 11-item checklist recommended by the Agency for Healthcare Research and Quality was used to assess the risk of bias in cross-sectional studies. Each study was judged as follows: low quality = 0-3, moderate quality = 4-7, and high quality = 8-11. The risk of bias in case-control studies was assessed by the Newcastle-Ottawa Scale. Article quality was judged as low quality = 0–5 stars, medium quality = 6-7 stars, and high quality = 8–9 stars.

### Data synthesis and analysis

We used Review Manager Software 5.3^®^ for statistical analysis. The data were analyzed by using the mean difference. We calculated the weighted mean difference (WMD) and associated 95% confidence interval (CI) for CDCD and CSND. Pooled estimates of effects were calculated by using random effects models. Heterogeneity was quantified by the *I*^2^ statistic; *I*^2^ > 50% defined high heterogeneity between studies. Subgroup analyses were performed when *I*^2^ > 50% to compare the heterogeneity as follows: country of study, type of IVCM, type of contact lens, CL wearing duration, and whether a daily change of CL.

## Results

### Search results

**FIGURE 1:**
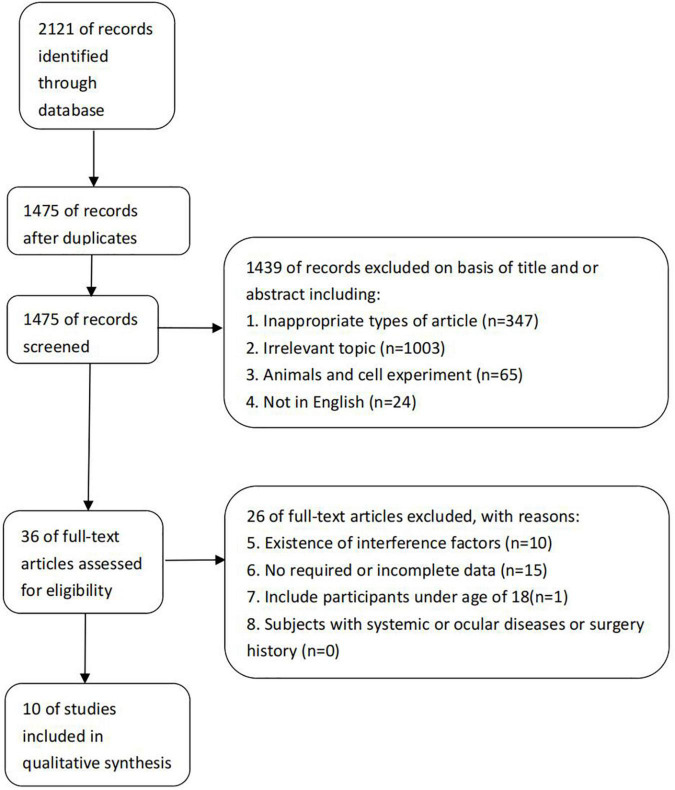
Flow diagram of the studies selection process.

After a systematic literature search, 2,121 references were identified, and 10 randomized clinical trials that met the inclusion criteria were included (Figure 1). The included studies consist of 2 case-control studies (11, 12) and 8 cross-sectional studies (5, 13–19). A total of 459 patients with 587 eyes were included. Details about the included studies are shown in Table 1.

**TABLE 1:** Characteristics of the included studies.

References	Eyes	Age (year)	Group	Quality	CDCD	CSND
Alzahrani et al. (11)	10	30 ± 5	Contact lens	Medium	**√**	
	10		Control			
Dogan et al. (13)	22	25.7 ± 8.2	Asymptomatic contact lens group	Moderate	**√**	
	28		Control			
Golebiowski et al. (14)	40	31.5 ± 5	Contact lens	Moderate		**√**
	40		Control			
Hu et al. (15)	16	27.3 ± 5.5	Asymptomatic contact lens group	Moderate		**√**
	16		Control			
López-De La Rosa et al. (5)	20	25 ± 5.19	Asymptomatic contact lens group	Moderate	**√**	**√**
	20		Control			
Lum et al. (16)	18	28 ± 10	Soft contact lens orthokeratology lens	Low		**√**
	18		Control			
Nombela-Palomo et al. (17)	35	24.8 ± 3.9	Orthokeratology lens	Moderate	**√**	
	21		Seefree			
	15		Control			
Saliman et al. (19)	20	31.1 ± 7.5	A2	Moderate	**√**	
	20		AO			
	20		Control			
Sindt et al. (18)	24	37 ± 14	Traditional hydrogel wearers	Moderate	**√**	
	82		Silicone hydrogel wearers			
	20		Control			
Tse et al. (12)	27	21.5 ± 3.9	Scleral lens	Medium	**√**	**√**
	27		Control			

CDCD, corneal dendritic cell density; CSND, corneal subbasal nerve density; A2, reusable Acuvue 2; AO, reusable Acuvue Oasys.

### Quality assessment

One trial was considered to have an overall high risk of bias. Nine studies were of moderate quality, although some uncertain risk of bias remained. Subgroup analysis for the outcome comparing low versus moderate quality trials was performed. More details are shown in Table 2.

**TABLE 2:** Subgroup meta-analysis of CSND.

Subgroup	Group by	No of studies	Eyes	Heterogeneity *I*^2^ (%)	WMD of CDCD (mm/mm^2^) (95% CI)	*P*-value for heterogeneity
Country of study	Western countries	4	196	87%	−3.15 (−7.68, 1.37)	0.007
	Asian countries	1	32	N	−2.11 (−4.66, 0.44)	N
Type of IVCM	HRTII/RCM	3	148	86%	−3.34 (−7.75, 1.07)	N
	HRTIII/RCM	2	80	82%	−2.28 (-9.13, 4.56)	0.1
Wearing duration	≤ 3 months	1	40	N	−6.24 (-11.66, -0.82)	0.26
	> 3 months	3	108	90%	−3.01 (−7.81, 1.78)	0.55

WMD, weighted mean differences; CDCD, corneal dendritic cell density; IVCM, *in vivo* confocal microscopy.

### Corneal dendritic cell density

**FIGURE 2:**
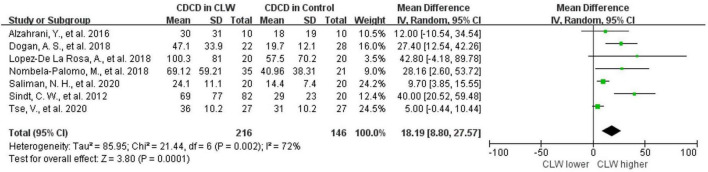
Forest plot of CDCD in CL wearer vs. control group. CI, confidence interval; IV, inverse variance; SD, standard deviation; CLW, contact lens wearers; CDCD, corneal dendritic cell density.

Seven studies were included in this analysis. CDCD was significantly higher in CL wearers than in controls, with an overall RR of 18.19 (95% CI 18.8–27.57, *p* = 0.0001), which supports the inflammatory ocular environment of CL wearers. The included studies showed high heterogeneity (*I*^2^ = 72%). The detailed results can be found in Figure 2. The subgroup analysis of the country and the type of contact lens also showed high heterogeneity. In the subgroup analysis of the type of IVCM, the HRTIII/RCM subgroup showed lower heterogeneity (*I*^2^ = 53%, *p* = 0.1). In the comparison grouped by wearing duration, the subgroups with a wearing duration of ≤3 months and >3 months both showed very low heterogeneity (*I*^2^ = 25%, *p* = 0.26 vs. *I*^2^ = 0%, *p* = 0.55). CL wearing time of more than 3 months significantly upregulated CDCD (RR 32.68, 95% CI 21.22–44.13, *p* < 0.0001). Daily contact lens replacement was also a source of heterogeneity. Both the daily disposable CL (*I*^2^ = 41%, *p* = 0.18) and daily reusable CL (*I*^2^ = 48%, *p* = 0.15) subgroups showed low heterogeneity. The daily disposable CL group (RR 32.68, 95% CI 21.22–44.13, *p* < 0.0001) showed a higher CDCD than the daily reusable CL group (RR 32.68, 95% CI 21.22–44.13, *p* < 0.0001). Further details are provided in Table 3.

**TABLE 3:** Subgroup meta-analysis of CDCD.

Subgroup	Group by	No of studies	Eyes	Heterogeneity *I*^2^ (%)	WMD of CDCD (mm/mm^2^) (95% CI)	*P*-value for heterogeneity
Country of study	Western countries	6	50	69%	15.87 (6.29, 25.45)	0.007
	Asian countries	1	312	N	27.4 (12.54, 42.26)	N
Type of IVCM	HRT/RCM	2	156	91%	21.2 (−13.01, 55.40)	0.0007
	HRTII/RCM	2	56	N	28.16 (2.6, 53.72)	N
	HRTIII/RCM	3	150	53%	17.24 (5.14, 29.33)	0.1
Type of CL	Silicone hydrogel	3	192	83%	23.96 (5.55, 42.38)	0.002
	Hydrogel	1	20	N	12.00 (−10.54, 34.54)	N
	orthokeratology lens	1	56	N	28.16 (2.6, 53.72)	N
	Scleral lens	1	54	N	17.21 (7.8, 26.62)	N
Wearing duration	≤ 3 months	4	170	25%	7.81 (3.93, 11.68)	0.26
	> 3 months	3	192	0%	32.68 (21.22, 44.13)	0.55
Daily change	Daily disposable CL	3	172	41%	27.72 (17.25, 38.18)	0.18
	Daily reusable CL	3	150	48%	7.68 (3.74, 11.61)	0.15

WMD, weighted mean differences; CDCD, corneal dendritic cell density; IVCM, *in vivo* confocal microscopy; CL, contact lens.

### Corneal subbasal nerve density

**FIGURE 3:**
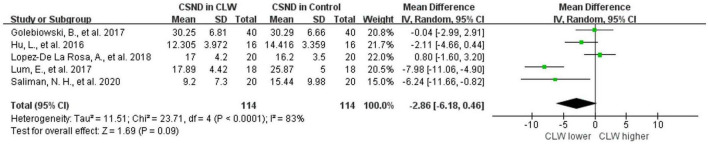
Forest plot of CSND in CL wearer vs. control group. CI, confidence interval; IV, inverse variance; SD, standard deviation; CLW, contact lens wearers; CSND, corneal sub-basal nerve density.

Five studies were included, and no significant difference was observed when assessing the change in CSND (*p* = 0.09), with an overall RR of −2.86 (95% CI -6.18–0.46). Wearing contact lenses did not significantly change CSND in the wearers compared to the controls. Included studies show high heterogeneity (*I*^2^ = 83%), and subgroup analysis did not reveal a source of heterogeneity. The details are shown in Figure 3 and Table 2.

## Discussion

As a highly prevalent treatment method, CL are receiving increasing attention. Although a significant number of CL wearers are diagnosed with DED, subclinical changes in the corneas of the wearers who are not clinically diagnosed remain of concern. When searching electronic databases, we found that several relevant studies had included subjects diagnosed with DED. This study focuses on the subclinical changes in healthy CL wearers. Thus, only studies investigating asymptomatic healthy CL wearers were selected. Ten articles were included in this study after screening. We analyzed CDCD, CSND, and assessed the effects of quality of the literature, country of study, type of IVCM, type of CL, wearing duration, and daily change on the outcomes.

Dendritic cells (DCs) are considered the most important resident corneal antigen-presenting cells (20). It has been demonstrated that corneal DCs are involved in multiple ocular surface diseases (21). The proliferation and maturation of DCs represent an inflammatory state of the ocular surface (22). Due to its real-time and non-invasive features, HRT/RCM of IVCM allows real-time observation of the corneal DCs from the cellular level.

Consistent with previous clinical findings, our overall results show a significant increase in CDCD in CL wearers (*p* = 0.0001). Our analysis demonstrated that even in asymptomatic healthy CL wearers, the cornea exhibited subclinical inflammation status. The changes, which were presented in asymptomatic healthy wearers, indicated that the inflammatory state was caused by CL, not CL-associated ocular surface diseases such as DED. Furthermore, we hypothesized that CL-induced subclinical inflammation was involved in the pathogenesis of DED in CL wearers. In subgroup analysis, the HRTIII/RCM subgroup showed lower heterogeneity compared to HRT/RCM and HRTII/RCM subgroups, suggesting that the results of studies using HRTIII/RCM for CDCD assessment are more stable and comparable.

The changes in the cornea have been associated with CL wear duration. A recent study showed that the density of corneal DCs significantly increased 1 week after soft CL wear, peaked at 1 month, and decreased after 3 months of CL wear (23). In our study, the duration of CL wear in the included studies ranged from 8 h to 14 years. We set 3 months as the boundary to divide the duration of wear into more than or less than 3 months. Unlike previous studies, we found that CL wearing for more than 3 months resulted in upregulated corneal DCs density compared with CL wearing for less than 3 months, and there were significant differences between the two groups. This study included different materials and types of contact lenses, and a comparison of wear duration for a particular type of CL may be more relevant.

It was generally considered that daily reusable contact lenses had a more significant impact on the cornea and carried a greater risk of disease than daily disposable lenses (24). However, our analysis demonstrated the opposite results. The CDCD of the daily disposable CL group was higher than the daily reusable CL group. Nonetheless, only 6 articles were included in this subgroup analysis, and more research may be needed to confirm this conclusion.

It has been demonstrated by IVCM and animal experiments that the density of DCs in the central cornea is lower than in the peripheral cornea (20). Five of the seven studies included did not clarify whether the DCs were in the central or peripheral cornea. Based on the analysis of their reported amounts, we speculated that these results may be from the peripheral cornea, and contacted the authors for confirmation. In two other studies, both central and peripheral cornea data were reported. To maintain data consistency, we only selected peripheral corneal data from these two studies for analysis.

The effects of CL wear on corneal nerves have been controversial. A few studies using IVCM have shown that wearing CL does not affect corneal nerve density, morphology, and distribution (8). Patel et al. (25) reported no reduction in corneal nerve density after CL wear, but corneal sensitivity decreased in CL wearers compared with the controls. Some studies presented conflicting results. Lum et al. (3) demonstrated by IVCM that CL wear resulted in decreased central corneal nerve density. Hiraoka et al. (26) reported that overnight orthokeratology lens wearing down-regulated corneal nerve density. Herein, we included five studies for the analysis of corneal nerve density. Two of these five studies reported a reduction in corneal nerve density, and the other three showed no significant changes. Our results showed that CL wear did not significantly alter the corneal nerve density. Although the results had no statistical significance, the CL group still showed lower corneal nerve density.

Subgroup analysis of the corneal nerve density was also performed, but the results did not reveal the source of heterogeneity and the differences between subgroups. This may be due to the small number of studies included. In addition to corneal nerve density, some studies have also investigated nerve fiber tortuosity, nerve fiber interconnections, the density of nerve branches, and nerve reflectivity. However, fewer than three studies reported these data. Therefore, the data were not further analyzed.

Our results showed that wearing CL did not directly lead to changes in the corneal nerves. Some studies have reported that CL wear reduced corneal sensitivity (27, 28). The decrease in corneal sensitivity has been attributed to disrupted corneal metabolism, mechanical effects, and sensory adaptations (29) caused by CL. In addition, CL wear can lead to the development of dry eye disease, further contributing to corneal nerve changes associated with the disease.

Our study has the following limitations: First, the number of articles included in this study was limited. If more new articles that meet the inclusion criteria can be included, more convincing outcomes might be drawn. Second, despite the attempt to control potential sources of heterogeneity, there were differences in study design, patient populations, and IVCM measurements that could have influenced outcomes. Meta-analysis that contains more studies with less heterogeneity may avoid the effect of heterogeneity on the results.

In conclusion, CL wear was associated with an increase in CDCD. When evaluating CDCD with IVCM, the results were more stable with HRTIII/RCM. CDCD was also related to CL wear duration and whether the lenses were changed daily. Although wearing contact lenses slightly reduced CSND, it was not statically significant. IVCM appears to be a feasible tool to assess subclinical changes in CL wearers.

## Author contributions

RG and KH conceived and designed the overall study. RG, JJ, YZ, and KH worked on data acquisition. RG and QL worked on data analysis and interpretation. RG and TC prepared the original manuscript. KH supervised and reviewed the manuscript. All authors read and approved the final version of the manuscript.
